# Precision Medicine in Neurodegenerative Diseases: Genomic Approaches to Target Amyloid-β, Tau, and Alpha-Synuclein Pathways

**DOI:** 10.2174/0113892029372437251010114053

**Published:** 2025-10-24

**Authors:** Zaid Waqar, Pranshul Sethi, Divya Jain, Kuldeep Singh, Omar Awad Alsaidan, Sami I. Alzarea, Jeetendra Kumar Gupta, Swati Saxena, Mukesh Chandra Sharma

**Affiliations:** 1 Department of Clinical Neurosciences, College of Medicine King Faisal University, Saudi Arabia;; 2 Chitkara College of Pharmacy, Chitkara University, Rajpura, Punjab-140401, India;; 3 Department of Pharmacology, College of Pharmacy, Shri Venkateshwara University, Gajraula, UP- 244236, India;; 4 Department of Microbiology, School of Applied and Life Sciences, Uttaranchal University, Dehradun-248007, Uttarakhand, India;; 5 Department of Pharmacology, Institute of Pharmaceutical Research, GLA University, Mathura, Uttar Pradesh, India;; 6 Department of Pharmaceutics, College of Pharmacy, Jouf University, Sakaka, 72341, Saudi Arabia;; 7 Department of Pharmacology, College of Pharmacy, Jouf University, Sakaka, Aljouf 72341, Saudi Arabia;; 8 IES Institute of Pharmacy, IES University, Bhopal, Madhya Pradesh, 462044, India;; 9 School of Pharmacy, Devi Ahilya Vishwavidalaya, Indore, M.P, India

**Keywords:** Precision medicine, neurodegenerative diseases, amyloid-β, tau, alpha-synuclein, genomic therapies, CRISPR-Cas

## Abstract

Neurodegenerative diseases, including Alzheimer’s and Parkinson’s disease, are characterized by the pathological aggregation of proteins such as amyloid-β, tau, and alpha-synuclein. These hallmark proteins play central roles in disease progression and represent promising targets for therapeutic intervention. Advances in precision medicine, driven by genomic technologies such as CRISPR-Cas systems, RNA-based therapies, and high-throughput sequencing, have enabled the development of tailored strategies to modulate these pathological pathways. This review examines the integration of genomic approaches in targeting amyloid-β, tau, and alpha-synuclein, emphasizing their potential to mitigate disease progression and improve patient outcomes. We highlight current progress in preclinical and clinical studies, discuss challenges associated with translating these therapies into clinical practice, and explore future directions for achieving therapeutic precision in neurodegenerative disorders. By examining the interplay of genetic, molecular, and therapeutic innovations, this review underscores the transformative potential of genomic medicine in addressing the unmet needs of neurodegenerative disease treatment.

## 1 INTRODUCTION

Neurodegenerative diseases are becoming increasingly prevalent as the population ages, resulting in higher mortality, greater health burdens, and increased costs for care, hospitalization, and medical treatment [1]. According to predictions, the proportion of Europeans aged 65 and older is expected to rise from 16% at present to 25% by 2030 [2]. Progressive degradation or dysfunction of neurons in specific brain and spinal cord regions is a hallmark of various age-related cerebral diseases collectively known as neurodegenerative disorders. Patients with these conditions often exhibit speech difficulties, movement impairments, and cognitive decline [3]. Dementia is the most common neurological disorder affecting cognitive function, currently impacting approximately 7 million people in Europe, with this number expected to triple by 2040. The disease typically persists for two to ten years [4, 5], during which patients require additional care and treatment to manage their challenges. Globally, more than $130 billion is spent annually on treating individuals with neurodegenerative diseases [6].

Many neurological conditions originate from the accumulation of misfolded proteins, which causes long-term neuronal damage and alters the body’s inflammatory response [7]. Understanding these common pathological mechanisms is critical for developing effective treatments in a field where no current pharmaceutical therapies can halt disease progression [7]. Existing therapeutic approaches primarily alleviate symptoms and temporarily slow disease progression, but do not address the underlying causes. Therefore, identifying accurate biomarkers of these diseases represents an essential step toward more effective interventions [7].

The field of precision medicine, also referred to as “personalized medicine” or “individualized medicine,” is rapidly advancing in both research and clinical practice. This approach emphasizes optimizing disease prevention and treatment rather than applying a uniform strategy to all patients [8]. By considering an individual’s unique genetics, biomarker profile, physiological characteristics, and behavioral factors, precision medicine aims to minimize adverse drug reactions and improve therapeutic outcomes [8]. Evaluating the molecular, environmental, and behavioral determinants of a disease enhances understanding of its pathogenesis, progression, and response to therapy. This approach facilitates more accurate diagnosis and enables the development of tailored, effective strategies for disease prevention and management [9].

The prevention and treatment of diseases across diverse populations increasingly rely on precision medicine. Many progressive neurological conditions, including glaucoma, age-related macular degeneration (AMD), and Alzheimer’s disease, affect multiple organ systems [9]. Disease progression may be influenced by neurological, endocrine, immune, cardiac, and digestive systems, with the factors impacting each system over a lifetime being highly variable. Moving beyond standard healthcare approaches, it is essential to develop improved diagnostic tools and individualized treatment plans to enhance healthcare inclusivity [10].

## 2 STUDY DESIGN AND METHODOLOGY

This review was conducted through a comprehensive and structured literature analysis focusing on genomic approaches targeting amyloid-β, tau, and alpha-synuclein pathways in neurodegenerative diseases. Peer-reviewed articles, clinical trial reports, and preclinical studies were identified from databases including PubMed, Scopus, and Web of Science using specific keywords such as “precision medicine,” “genomic therapies,” “CRISPR-Cas9,” “RNA-based therapies,” and the target protein names. Inclusion criteria comprised original studies, systematic reviews, and meta-analyses published in English that provided mechanistic insights, therapeutic strategies, or clinical outcomes related to these proteins. Data extraction emphasized molecular mechanisms, genomic intervention strategies, preclinical efficacy, and clinical trial status. Particular attention was given to studies elucidating the interplay among amyloid-β, tau, and alpha-synuclein. Emerging genomic technologies, such as CRISPR-Cas systems, antisense oligonucleotides, small interfering RNAs, and aptamers, were also evaluated for their therapeutic potential. The findings were synthesized thematically to highlight current advancements, translational challenges, and future research directions.

## 3 PATHOLOGICAL MECHANISMS OF AMYLOID-β, TAU, AND ALPHA-SYNUCLEIN

According to the updated amyloid cascade theory, the primary cause of neuronal damage and the impairment of other cell types is the diverse and dynamic forms of soluble Aβ (amyloid-β) oligomers (AβO) [11]. Despite differences in their amino acid sequences, amyloid oligomers share a common structural appearance, suggesting that the pathogenic mechanisms underlying all amyloid-associated diseases are similar. Lower molecular weight structures, such as oligomers, exhibit greater structural variability [12]. A wealth of research indicates that the size of Aβ assemblies influences their neurotoxicity. Both Alzheimer’s disease patients and genetically modified mouse models have been shown to harbor different types of soluble AβO in the brain, reflecting this size diversity [12].

Proto-fibrillar Aβ and synthetic Aβ oligomers (AβO) induce endoplasmic reticulum (ER) stress, increase oxidative stress, trigger neuroinflammation, and cause synaptic and neuronal hyperactivity, often culminating in neuronal death [13]. Proto-fibrillar Aβ has been observed in the brains of genetically modified mice exhibiting Alzheimer-like symptoms as well as in patients with Alzheimer’s disease. Furthermore, AβO was shown to impair memory function in these mouse models [14].

Numerous pathways, including direct interactions with cell membranes, associated structures, and specific receptors, may contribute to AβO-induced neurotoxicity. Although much is known about aggregated Aβ and its role in Alzheimer’s disease, the specific pathogenic forms of Aβ in other neurological conditions remain less well understood [15]. Tau oligomers (TauO), derived from both laboratory preparations and brain tissue, are increasingly recognized as highly neurotoxic. *In vitro* and *in vivo* studies indicate that TauO can promote intracellular tau accumulation, disrupt neural connectivity, and impair memory [16]. Laboratory experiments have shown that TauO isolated from the brains of patients with traumatic brain injury, dementia with Lewy bodies, progressive supranuclear palsy, and Alzheimer’s disease can seed the formation of tau monomers [17]. Particular tau species in Alzheimer’s patient brains have been linked to disease severity [3].

Variations in tau accumulation and metabolism suggest that disease manifestation may differ among individuals. Behavioral changes were observed in normal mice following intracerebral injection of TauO, which spread from the injection site to other brain regions [18]. Similar to prion proteins, tau can propagate between neighboring neurons, as evidenced in studies of human Alzheimer’s brains and animal models. However, the mechanisms governing tau uptake and release between adjacent cells remain incompletely understood [19].

Several mechanisms mediate α-synuclein (α-Syn) propagation, including membrane sensors, exosomes, ectosomes, and tunneling nanotubes (TNTs). According to the α-Syn cascade hypothesis, α-Syn forms aggregates well before the onset of clinical symptoms [20]. The precise factors driving α-Syn aggregation *in vivo* remain unclear, although laboratory studies indicate that conditions such as pH and ionic strength can influence aggregation. Multiple conformations of α-Syn, collectively referred to as α-Syn oligomers (α-SynO), have been extensively characterized in recent reviews [20].

The neurotoxic effects of α-SynO and their mechanisms of propagation in various neurodegenerative diseases have also been investigated. α-SynO can impair mitochondrial and nuclear function, disrupt synaptic activity, disturb proteostasis, induce endoplasmic reticulum stress, and trigger neuronal death. Both laboratory-generated α-SynO and those spontaneously arising in TgM83 mice have been shown to induce α-Syn pathology in specific cell types and brain regions [21].

Clusters of α-synuclein (α-Syn) are associated with dysfunction in both neurons and astrocytes, the brain’s supporting glial cells. Disorders such as Parkinson’s disease, dementia with Lewy bodies, and, occasionally, multiple system atrophy are characterized by α-Syn aggregation [22]. The accumulation of α-Syn aggregates impairs autophagy and lysosomal degradation, leading to mitochondrial dysfunction in astrocytes [23]. Several mechanisms have been proposed for α-Syn–mediated neurotoxicity, including tunneling nanotubes (TNTs), exosomes, and specific cellular membrane sensors, although the precise pathways remain incompletely understood [24].

Protein aggregates often coalesce to form heterogeneous assemblies across various diseases, suggesting potential molecular interactions among them. Although recent studies indicate links between tau, α-synuclein (α-Syn), and Aβ, the precise mechanisms by which these proteins interact and contribute to disease remain unclear [25]. The phenomenon of cross-seeding is particularly important when analyzing the interplay among these three proteins. This section focuses on the *in vitro* and *in vivo* cross-seeding of tau, α-Syn, and Aβ, emphasizing their interactions under experimental and physiological conditions [26]. In Alzheimer’s disease, the relationship between tau and Aβ is well established. For instance, we previously demonstrated a negative interaction between Aβ and tau by showing that TauO-targeted passive immunotherapy ameliorated cognitive deficits in a mouse model of Alzheimer’s disease [27].

As Alzheimer’s disease progresses through multiple stages, simulation modeling indicates that tau propagation occurs more rapidly in brain regions with elevated amyloid-β (Aβ). Soluble Aβ oligomers (AβO) can activate extrasynaptic glutamate receptors, particularly N-methyl-D-aspartate receptors (NMDARs), leading to the activation of AMP-activated protein kinase (AMPK) at the synaptic level [28]. AMPK subsequently phosphorylates dendritic tau, causing its detachment from microtubules and promoting aggregation. At the proteome level, AβO significantly impacts tau, TAR DNA-binding protein 43 (TDP-43), and heterogeneous nuclear ribonucleoproteins in human-induced pluripotent stem cells [29].

The direct relationship between APOE4 and Aβ, along with the activation of specific kinases, may contribute to the indirect interactions between tau and Aβ [30]. Aβ and tau cooperate to impair mitochondrial function, leading to neuronal damage in Alzheimer’s patients and disrupting brain circuits in animal models, as illustrated in Fig. (**1**). Furthermore, studies revealing genetic links between tau and α-synuclein (α-Syn), as well as the presence of their fibrillar aggregates in various neurodegenerative diseases, suggest a potential association between these two proteins [31].

The co-occurrence of α-synuclein oligomers (α-SynO) and tau oligomers (TauO) derived from Parkinson’s disease brains exacerbated tau-related pathology and cognitive deficits in a mouse model of tauopathy more than TauO from progressive supranuclear palsy (PSP) brains alone [32]. In TgM83 mice with synucleinopathy, tauO-targeted passive immunotherapy worsened cognitive and motor deficits. Another study linked tau pathology to hippocampal memory and synaptic abnormalities in TgG2.3 synucleinopathy mice [33], whereas tau clearance ameliorated synaptic dysfunction and cognitive impairments associated with α-Syn TgA53T [34].

Comorbidities are common in over 50% of Alzheimer’s disease patients, with vascular dementia and dementia with Lewy bodies being the most prevalent [35]. Hybrid aggregates of Aβ and α-Syn have been observed in certain genetically modified mice and in the brain tissues of individuals with Parkinson’s and Alzheimer’s diseases, suggesting a direct interaction between these proteins [34]. In 5xFAD mice, which exhibit extensive Aβ plaque deposition, the introduction of preformed α-Syn fibrils accelerated α-Syn pathology, indicating that Aβ deposits may exacerbate α-Syn pathology in a feed-forward loop [36]. Furthermore, the abundance of Aβ plaques in two TgAPP mouse models was correlated with the presence of either wild-type α-Syn (α-SynWT) or mutant α-SynA30P [37].

Recent *in vitro* studies demonstrating that Aβ and α-synuclein (α-Syn) co-aggregate and interact directly *via* the C-terminal region of Aβ support these *in vivo* findings [38]. Both monomeric and oligomeric α-Syn were found to inhibit Aβ fibrillation while promoting its oligomerization. Molecular dynamics simulations further suggest that the two proteins can combine to form hybrid complexes [39]. Moreover, the aggregation of α-Syn in neuronal cells was facilitated by the introduction of low concentrations of Aβ42. Aβ also induces post-translational modifications of α-Syn through enzymes such as cyclin-dependent kinase 5 (Cdk5), casein kinase 2, and polo-like kinase 2 (PLK2) [40].

Brains of individuals with Parkinson’s disease (PD), Parkinson’s disease dementia (PDD), and dementia with Lewy bodies (DLB), as well as cerebrospinal fluid from Alzheimer’s disease (AD) patients, exhibit concurrent abnormalities in tau and Aβ [41]. Co-oligomers containing tauK18, Aβ, and α-Syn are more likely to form than α-Syn self-oligomers. This has been demonstrated using statistical modeling and single-molecule two-color coincidence detection. Due to the interactions among these proteins, heterogeneous protein assemblies may exist within a single disease or across multiple neurodegenerative disorders [42].

In addition to tau pathology, Aβ, α-synuclein (α-Syn), and TDP-43 have been detected in Pick’s disease (PiD), corticobasal degeneration (CBD), and progressive supranuclear palsy (PSP). In most Alzheimer’s disease cases, tau pathology coexists with either TDP-43 or α-Syn abnormalities [13]. In dementia with Lewy bodies (DLB) and multiple system atrophy (MSA), Aβ pathology is more prevalent than Aβ co-occurring with TDP-43. In amyotrophic lateral sclerosis (ALS) and frontotemporal dementia (FTD) with TDP-43 inclusions, the frequencies of Aβ and α-Syn pathologies are comparable to those of TDP-43 pathology [13].

Advances in imaging techniques and fluid biomarkers for predicting health outcomes will be discussed in the following sections, along with their role in improving diagnosis [43]. Given the complexity of neurodegenerative diseases, which involve multiple factors and protein-related challenges, combination therapy is considered a rational approach for their management [43]. Immunotherapy targeting α-synuclein (α-Syn) has been proposed due to its involvement with tau, Aβ, prion, and TDP-43 proteins. Individuals with the autosomal-dominant E280A mutation in Presenilin1 (NCT01998841) are participating in a phase II trial of Crenezumab focused on β-amyloid and tau [44, 45].

The impact of Crenezumab on tau levels over time in the same participants is being assessed in a second phase II trial (NCT03977584) [46]. These studies exemplify an innovative strategy that combines immunotherapies targeting multiple key proteins with biomarkers and imaging for disease prediction. Investigations into the composition and activity of pathogenic α-Syn aggregates underscore the importance of understanding their mechanisms to develop effective therapeutic interventions.

## 4 GENOMIC TECHNOLOGIES IN PRECISION MEDICINE

Genomic medicine, a promising field, leverages an individual’s genetic information to optimize medical care. This approach facilitates the prediction of health risks, disease prevention, early detection, and the provision of personalized treatments [47]. The concept originates from the National Human Genome Research Institute (NHGRI, USA). By enabling precise diagnosis, improved risk assessment, screening-based prevention, and tailored therapeutic strategies, genomic medicine has the potential to transform healthcare for both common and rare disorders [48].

By investigating the genetics of numerous diseases, researchers have rapidly bridged basic science with clinical research, ushering in a new era of healthcare. Genomic medicine, by reducing preventable risks and enhancing health outcomes, strengthens primary care [49]. Precision or personalized medicine is poised to take a leading role in healthcare practices. Although its applications are broad, the development of precision medicine largely depends on advances in genomic technologies. The increasing demand for robust clinical data to guide treatment decisions for common diseases has further emphasized the integration of genomic medicine into patient care, thereby improving the quality of evidence-based practice [50].

Genetic medicine has advanced from Mendel's work to modern next-generation sequencing (NGS) technology, and the Human Genome Project in 2003 was a major milestone in this field, although it is only one of many achievements in its development. Instead of marking the end of an era in genetics, the Human Genome Project's (HGP) success marked the beginning of a new one [51]. Currently, many doctors are using high-throughput genetic testing. The use of genomic medicine has been improved by recent developments in high-throughput technologies, such as genome-wide association studies (GWAS) and next-generation DNA sequencing. These advances help to better manage a range of ailments, from mild genetic issues to more serious ones [51].

Whole exome sequencing (WES) is used to determine the cause of health problems in individuals with undiagnosed diseases. Advances in genetic medicine have significantly improved the treatment of common diseases, including cancers and heart conditions, through precision medicine [52]. Significant progress in pharmacogenetics and pharmacogenomics has been made by analyzing genetic variations. Gene editing techniques are considered difficult but effective tools for enabling treatments. Several studies have explored new ways to increase the effectiveness of gene editing and reduce side effects [53]. CRISPR-Cas9, RNA therapies, and high-throughput sequencing are approaches that have revolutionized gene editing [54].

### 4.1 CRISPR Cas-9

Most bacteria and archaea have an adaptive immunity mechanism called CRISPR-Cas that protects them from viruses, phages, and other foreign genetic entities. It is composed of CRISPR repeat-spacer segments that can be processed into trans-activating CRISPR RNA (tracrRNA) and CRISPR RNA (crRNA) [55]. The Cas proteins, which are enzymes that can cleave DNA, are encoded by CRISPR-associated (cas) genes. Cas proteins can cleave foreign DNA into short fragments when prokaryotes encounter it [55]. These fragments are then incorporated as unique spacers into the CRISPR array. When the same invader attacks again, crRNA rapidly recognizes and binds to the foreign DNA, protecting the host by enabling the Cas protein to cut it [56].

There are two main classes (Class 1 and Class 2) of CRISPR-Cas systems, along with six types (I to VI) and numerous subtypes. In contrast to Class 2 systems (types II, V, and VI), which use a single protein in their functional complexes, Class 1 systems (types I, III, and IV) use multiple proteins [57]. Among the most extensively studied and widely used CRISPR-Cas systems is the Type II CRISPR-Cas9 system, which is derived from Streptococcus pyogenes (SpCas9) [58]. The two main components of the CRISPR-Cas9 system are the Cas9 enzyme, which cleaves DNA, and single-guide RNA (sgRNA), a short RNA segment that directs Cas9 to the target site in the DNA. The two DNA-cleaving domains of the Cas9 protein are called HNH and RuvC, with each domain cleaving one strand of the double-stranded target DNA [59].

The Cas9 enzyme collaborates with a single-guide RNA (sgRNA), which is a condensed form of the natural RNA molecules tracrRNA and crRNA [56]. A complex known as a ribonucleoprotein (RNP) is formed when sgRNA and Cas9 work together to recognize and cleave specific DNA sequences. For this to occur, a unique short sequence called the protospacer adjacent motif (PAM), which helps Cas9 identify and bind to the correct location, must be present in the DNA [56].

Once Cas9 is guided to the target by the sgRNA, it cuts both strands of the DNA, generating a double-strand break (DSB). Cells can repair this break *via* two main pathways. The faster method, known as non-homologous end joining (NHEJ), operates during most phases of the cell cycle [59]. Although error-prone, it does not require a matching DNA template. It often results in small insertions or deletions (indels), which can shift the reading frame or create premature stop codons, thereby inactivating the gene [60, 61]. A more precise pathway, homology-directed repair (HDR), uses a matching DNA template to repair the break, enabling accurate gene editing. Scientists can also employ multiple sgRNAs simultaneously to delete large DNA regions or inactivate several genes [54].

### 4.2 RNA-based Therapies

#### 4.2.1 Antisense Oligonucleotides

Short DNA or RNA strands known as antisense oligonucleotides (ASOs) are designed to bind to specific RNA sequences in cells and modulate how those RNAs are processed or translated. Although their sequence primarily determines their structure, scientists can also chemically modify them to enhance their stability and targeting accuracy [62]. In 1998, the FDA approved fomivirsen, the first ASO drug. It was used to treat CMV retinitis, an eye disease that can affect HIV-positive individuals. By binding to CMV mRNA, fomivirsen prevents the synthesis of immediate-early 2, a protein essential for viral replication [63]. However, the FDA withdrew fomivirsen from the market in 2001 due to declining demand and the availability of newer HIV medications [64].

Although ASOs can act through multiple mechanisms, approved drugs that use them typically fall into one of two categories based on their mode of action. The first group functions by binding to a specific region of the target mRNA and inducing its degradation, thereby preventing the synthesis of undesirable or harmful proteins [62].

A common modification for antisense oligonucleotides (ASOs) is to flank a core DNA segment with chemically modified RNA. A unique enzyme, RNase H, can recognize the DNA-RNA hybrid formed when these ASOs bind to their target RNA [65]. The target RNA is then degraded as RNase H cleaves the RNA strand at the site paired with the DNA. This process occurs in both the cytoplasm and nucleus of the cell [66]. One advantage of this strategy is that it can more effectively target noncoding RNA elements compared to siRNA-based therapies, which are primarily active in the cytoplasm [67].

ASOs are currently being investigated in several clinical studies as potential therapies for severe neurological conditions. For example, IONIS-HTTRx is being evaluated for Huntington's disease because it targets the mutant huntingtin protein, and tofersen is being studied for familial amyotrophic lateral sclerosis (ALS), which is caused by mutations in the SOD1 gene [64, 68]. According to preliminary findings, ASO-based therapies may offer promising new avenues for the treatment of complex disorders.

#### 4.2.2 Small Interfering RNAs

siRNAs regulate the expression of target RNAs by utilizing the body's natural RNA interference mechanism. The Argonaute (Ago) protein and small RNA molecules combine to form the RISC complex in endogenous RNA interference. By binding to specific target mRNAs based on their sequence, this complex inhibits their translation [69]. Patisiran, givosiran, and lumasiran are three siRNA-based medications approved by the US FDA. The suffix of these medications is usually “-siran.” siRNA-based therapeutics employ a similar mechanism to achieve this effect [70]. After being incorporated into the RISC complex, siRNA duplexes integrate into the endogenous RNAi pathway. During this process, the passenger strand is released, while the guide strand remains bound to the Ago protein. The guide strand then directs RISC activity toward the target [71].

Synthetic or chemically modified double-stranded RNA molecules are introduced into the body as part of siRNA therapy. These molecules are complementary to the specific mRNA that needs to be silenced [70]. The siRNA enters the cell and is incorporated into the RISC complex. The guide strand remains bound to the Ago protein, while the passenger strand is cleaved. This enables RISC to locate and degrade the corresponding mRNA, thereby halting the synthesis of the corresponding protein [70].

Advanced delivery mechanisms are often employed in siRNA pharmaceutical formulations to ensure stability and effective cellular uptake. Lipid nanoparticles (LNPs) are commonly used to protect siRNA from degradation and facilitate its entry into target cells [70]. To achieve efficient and targeted gene silencing, all FDA-approved siRNA therapeutics such as patisiran for hereditary transthyretin amyloidosis, givosiran for acute hepatic porphyria, and lumasiran for primary hyperoxaluria type 1 utilize specialized delivery platforms [70].

#### 4.2.3 Aptamers

Aptamers are genetically engineered strands that bind to specific proteins and modulate their function. To date, only one RNA-based aptamer drug has received FDA approval [72]. Aptamers are identified using the suffix “apt.” Pegaptanib, a 28-nucleotide molecule, is modified with two polyethylene glycol (PEG) groups at its end. Its purpose is to bind vascular endothelial growth factor (VEGF) isoform 1 [73]. By preventing VEGF from binding to its receptor, pegaptanib reduces cellular proliferation, which is the main effect of VEGF signaling [74]. This aptamer was developed as a treatment for wet age-related macular degeneration (AMD) [75].

Exogenous mRNAs that can be translated into functional proteins are used in messenger RNA-based therapies. To facilitate recognition by the cellular translational machinery, a cap analog is attached to the 5′ end of the RNA during the *in vitro* transcription process [76]. The delivery of mRNAs is complicated by the fact that long RNA sequences can trigger immune responses through Toll-like receptors (TLRs), which has limited their therapeutic use, especially in early stages [77]. mRNA-based treatments and aptamers for targeting neurodegenerative disorders in precision medicine and therapeutic applications are summarized in Table **1** [78].

Exogenous mRNAs are introduced into cells in the first category to supplement or replace the proteins that the cells normally produce. For example, mRNA therapy can help individuals with a hereditary condition that causes a deficiency of a vital enzyme by restoring enzyme levels [101]. To address the MUT enzyme deficiency, a research team administered mRNA encoding the enzyme to mice with methylmalonic acidemia. The second category includes mRNAs designed to function as vaccines for cancer or infectious diseases [102]. Examples include the mRNA rabies vaccine mentioned earlier and several mRNA vaccines under development for RNA viruses such as influenza. Furthermore, mRNA-based therapies are expected to be highly effective in treating hereditary disorders caused by protein deficiencies, indicating potential for future advancements and optimization of these therapies [101].

## 5 TARGETING AMYLOID-β PATHWAYS USING GENETIC APPROACHES

The progressive breakdown of a larger protein called APP produces Aβ, making this process a central focus in Alzheimer's disease research. APP is a transmembrane protein consisting of 695–770 amino acids [103]. It can be cleaved by either BACE1 or α-secretase into two smaller fragments: CTFβ, which is 99 amino acids long, and CTFα, which is 83 amino acids long. The membrane-associated enzyme γ-secretase further processes CTFβ into a 4 kDa Aβ fragment and CTFα into a smaller 3 kDa component called P3 or Aα [104].

Additional Aβ-like fragments have been reported, but they are considered less significant. APP processing has been reviewed extensively in previous studies [105]. A key aspect of regulating these events is the compartmentalization of processing enzymes. α-Secretase and BACE1 operate in different cellular locations [105]. APP processing is not inhibited by BACE1, although its contribution is relatively small. Elevated APP levels lead to increased production of CTFβ and Aβ [106].

Inhibition of α-secretase does not increase Aβ synthesis. This indicates that α-secretase does not block Aβ production, even though it cleaves within the Aβ sequence and plays a critical role in APP processing. Both constitutive and regulated secretion pathways generate Aβ, so the cellular components involved vary depending on the cell type [107]. When APP reaches the cell membrane, it is processed into CTFα either through the secretory pathway or by constitutive or active forms of α-secretase. γ-Secretase, located in the Golgi apparatus, cell surface, and other cellular compartments, then cleaves the membrane-associated CTFα fragment [108].

An APP that cannot be cleaved by α-secretase is internalized into small cellular compartments called endocytic vesicles. Within these vesicles, the enzyme BACE1 cleaves APP to generate CTFβ. BACE1 exhibits optimal activity under acidic conditions [109]. Aβ40 and Aβ42 are subsequently produced by γ-secretase, transported to the cell surface, and released *via* recycling vesicles [109]. Synaptic dysfunction is thought to arise from interactions between phosphorylated MAPT and accumulated Aβ, which form oligomers [28]. The development of dementia is influenced by environmental factors, genetics, and epigenetics, as well as lifestyle factors such as poor nutrition, lack of physical activity, and early exposure to metals and pesticides, as described in the “LEARN” model [110].

Changes in the APP, PS1, and PS2 genes cause a variety of problems, even though familial Alzheimer's disease (FAD) accounts for only 5% of Alzheimer's cases [111]. These problems begin with the accumulation of Aβ, which forms sticky aggregates in the brain. This process can lead to dementia, brain atrophy, neuronal loss, and damage to neural connections [111]. Although there is strong evidence linking Aβ to Alzheimer's disease in terms of toxicity and genetics, some researchers still question its significance, considering it a secondary issue [3]. Most discussions focus on the relationship between Aβ toxicity and cognitive decline in Alzheimer's disease, as well as its potential as a therapeutic target. These ideas have gained broad acceptance following the failure of several Aβ-targeted therapies [112].

*In vivo* tagging of Aβ suggests that these pathological changes could begin as early as 20 years before dementia symptoms appear. This indicates that dementia develops in stages, and Aβ could serve as a useful early biomarker, similar to how cholesterol levels are monitored for heart disease [113]. Imaging studies support the idea that certain proteins and brain structures show signs of disease before symptoms manifest. Cerebral impairment may be present even before mild dementia develops [114]. For individuals at higher risk, interventions aimed at reducing specific harmful substances in the brain should be initiated as early as possible to delay the onset of dementia. The concept of proactive or preemptive intervention is increasingly recognized and discussed today [114].

These findings highlight the need to understand the fundamental mechanisms underlying dementia development and the multiple stages that may require intervention for preventive treatments. Effective approaches to studying the disease include imaging techniques to visualize its features, genetic analyses to identify minor genomic variations associated with it, and genomic and proteomic methods to detect disease-related alterations [115]. Following the discovery of three major mutations in familial Alzheimer's disease (FAD), researchers have actively searched for genes associated with AD and other neurological disorders, leading to the identification of numerous disease-linked genes [116]. These findings also suggest that several neurodegenerative diseases could be caused by specific gene mutations alone. Notably, APP, PS1, and PS2 are all involved in Aβ synthesis, while only a few variants are linked to Aβ degradation, as illustrated in Fig. (**2**) [117].

According to recent research, blocking γ-secretase increases Aβ levels unexpectedly by preventing the degradation processes that normally break it down. It remains to be determined whether PS1 and APP variants that reduce γ-secretase activity could have a similar effect [118]. Recent studies also indicate that amyloid-beta (Aβ) and MAPT accumulation in Alzheimer's disease (AD) occurs alongside many other proteins, suggesting that maintaining proper protein homeostasis is a challenge associated with the disease [119]. Since the completion of the Human Genome Project, numerous Genome-Wide Association Studies (GWAS) have identified multiple genetic risk factors linked to AD [119].

Genome-wide association studies have identified ApoE-ε4 as a significant risk factor for Alzheimer's disease. ApoE-ε4 has modest penetrance but a strong association with typical late-onset forms of the disease [120]. While ApoE has been linked to familial hypercholesterolemia syndromes in the periphery, it is synthesized by glial cells in the brain and normally regulates cholesterol and triglyceride homeostasis [120]. Through several mechanisms, including reduced Aβ clearance, ApoE, the primary cholesterol transporter in the brain, appears to play a major role in Alzheimer's disease (AD). ApoE exists in three isoforms: APOE2, APOE3, and APOE4 [121].

Alzheimer's disease is more likely to occur in individuals who carry the ε4 allele, and the risk increases with the number of copies. The ε4 variant may be less protective than the ε2 variant [122]. According to Pittsburgh compound B-based brain imaging studies, middle-aged, cognitively healthy individuals with the ApoE-ε4 gene have significantly higher brain amyloid levels than those without this gene. This is often accompanied by lower cerebrospinal fluid concentrations of Aβ42 [123].

Studies show that centenarians have a much lower prevalence of the ApoE-ε4 gene, which is associated with early-onset cardiovascular disease, compared to non-centenarians. Conversely, individuals with longer lifespans tend to have higher levels of the ApoE-ε2 gene, which is linked to certain types of high cholesterol. ApoE-ε4 is a complex risk factor for human diseases [124]. By providing protection against age-related macular degeneration, a condition associated with amyloid accumulation in ocular deposits called drusen, it appears to benefit some individuals. Multiple hypotheses exist regarding the role of ApoE-ε4 [125].

One theory suggests that ApoE-ε4 alters the balance of cholesterol in the brain, which could promote Aβ aggregation and lead to plaque buildup. It is also thought to reduce the efficiency of Aβ degradation [126]. ApoE-ε4 may be incorporated into plaques in smaller fragments. Ultimately, this can contribute to neurodegeneration associated with Alzheimer's disease by impairing APP processing and affecting neuronal survival [126]. Evidence indicates that ApoE facilitates Aβ aggregation into amyloid fibrils. However, the presence of the ApoE-ε4 variant reduces the efficiency of this ApoE-mediated pathway for Aβ fibril formation [126].

Alzheimer's disease (AD) occurs in about 1% of families due to genetic mutations. While CRISPR/Cas9 gene editing may help treat familial Alzheimer's disease (FAD), it is unlikely to have a major impact on sporadic Alzheimer's disease (SAD) [127]. Since Aβ metabolism dysfunction is linked to both familial and sporadic forms of the disease, reducing Aβ production could be a useful therapeutic strategy regardless of type [128]. Because most cases of Alzheimer's disease are sporadic with unknown causes, using CRISPR/Cas9 as a treatment may not be broadly applicable [128]. Less than 1% of AD patients have specific genetic changes that increase APP synthesis, which promotes Aβ production [129]. Although these mutations account for a small proportion of cases, they lead to elevated Aβ levels. Early-onset Alzheimer's disease is caused by mutations in the presenilin 1 (PSEN1) and presenilin 2 (PSEN2) genes, which accelerate the production of Aβ1-42 by altering APP cleavage [116].

Many of these mutations are indicative of early-onset AD, defined as onset before 60 years of age. These autosomal dominant mutations can be significantly corrected using CRISPR/Cas9 technology [130]. Recent studies have demonstrated that similar mutations can be corrected by this gene-editing approach. For instance, the PSEN2N141I mutation was corrected in neurons derived from patient stem cells using CRISPR/Cas9 [131]. This correction restored the balance between Aβ42 and Aβ40 levels [132]. Furthermore, electrophysiological deficits were substantially improved after the PSEN2 mutation was corrected using this strategy [133].

Previous studies using patient-derived iPSCs and CRISPR/Cas9 to correct PSEN gene mutations in familial Alzheimer's disease (FAD) reported comparable findings. Another study demonstrated that this approach effectively eliminated Swedish APP mutations in fibroblasts derived from patients, resulting in a 60% reduction in Aβ levels. The Swedish variant rapidly binds to the β-secretase site of APP [131].

In Tg2576 mice, which carry multiple APP Swedish mutations, researchers also targeted this mutation. This was achieved by injecting DNA containing both Cas9 and guide RNAs *via* AAV vectors into the hippocampal regions of the transgenic mice. Post-injection changes included minor one-base-pair insertions in the APP Swedish gene. Only 2% of the genes at the injection site were affected when CRISPR/Cas9 was directly administered into the hippocampus. Since each neuron in Tg2576 mice contains approximately 100 copies of the transgene, the delivered amount of CRISPR/Cas9 was insufficient to fully correct the Swedish mutation [134].

This finding indicates that a more detailed examination of specific hippocampal cells is necessary to optimize editing efficiency and evaluate its function *in vivo* [135]. Researchers have also demonstrated that modifying the terminal region of the APP gene can reduce β cleavage and Aβ production, providing a way to assess the effectiveness of this gene-editing approach for sporadic Alzheimer's disease (SAD). This modification prevents further interactions with BACE1 in endosomes, thereby blocking a crucial step in Aβ synthesis [136].

Research using human iPSC-derived neurons, cultured neurons, cell lines, and mouse brains showed that this reduces the physical interaction between APP and BACE1, which in turn lowers Aβ production [137]. One important risk factor for late-onset Alzheimer's disease is the APOE4 gene. APOE exists in three isoforms: APOE2, APOE3, and APOE4. At positions 112 and 158, arginine is substituted for cysteine, making each isoform differ by only a single amino acid. Their functional characteristics vary accordingly [138]. One copy of the rare E2 variant of APOE can reduce the risk of Alzheimer's disease by up to 40%. The common APOE3 variant does not appear to significantly influence AD risk. In contrast, APOE4, present in approximately 10–15% of the population, increases the risk of developing Alzheimer's disease and lowers the age of onset [139].

A single copy of the E4 gene (E3/E4) can increase the risk of Alzheimer's disease threefold, while two copies (E4/E4) can increase the risk by approximately ten to fifteen times. At least one APOE4 allele is present in 65–80% of individuals with Alzheimer's disease [140]. Although many of APOE4's detrimental effects are linked to Aβ accumulation, a recent study indicates that it may also enhance Tau phosphorylation in neurons derived from human stem cells, independent of Aβ levels [141]. To prevent APOE4-associated pathology in the model system, this study employed zinc-finger nuclease gene editing to convert APOE4 into APOE3. CRISPR/Cas9 could serve as an effective tool for converting APOE4 into either APOE2 or APOE3 [133, 141].

A study highlights the key structural differences between APOE3, APOE4, and APOE2. These differences are related to the interaction between two amino acids, Arg-61 and Glu-255, across a salt bridge [142]. By using CRISPR/Cas9 to modify one of these amino acids, it may be possible to reduce the risk associated with the APOE4 gene [143]. Successfully treating Alzheimer's disease through non-viral CRISPR/Cas9 delivery to the brain presents several challenges [144]. To effectively deliver the therapeutic payload to the desired site, the vectors must be stable. Once inside the target cells, the vectors must be internalized to reach the nucleus and avoid lysosomal degradation [145].

## 6 TAU-DIRECTED GENOMIC THERAPIES

Tau plays several important roles in brain function. Its primary function is to facilitate binding to microtubules. Microtubules are structures that support various cellular processes, including the development of nerve fibers, and help maintain cellular stability [146]. Tau consists of several regions, including the proline-rich region, the microtubule-binding repeat (MTBR) domain, the C-terminus, and the N-terminal projection domain, each with distinct roles and interaction mechanisms [147]. In humans, there are six main isoforms of the tau protein. These isoforms differ based on the number of MTBR domains (either 3R or 4R) and the number of repeats in the protein's N-terminal segment (0N, 1N, or 2N). These differences arise from the inclusion or exclusion of exons 2 or 3 (0, 1, or 2N) and exon 10 (3R or 4R) [148]. The most common tau isoform in fetuses is 3-repeat tau (3R tau) [148].

The amounts of 3R and 4R tau are roughly equal in adulthood. The tau protein variant that accumulates in the brain can be used to classify tauopathies: 3-repeat (as in Pick's disease), 4-repeat (as in Progressive Supranuclear Palsy), and a combination of both 3-repeat and 4-repeat (which includes most secondary tauopathies) [149]. Two tau variants remain poorly understood. “Big tau,” generated after the N-terminal domain, produces an extra sequence of approximately 250 amino acids by encoding exon 4a [150]. Most big tau is found in the adult peripheral nervous system and certain brain neurons. Another unique variant incorporates exon 6 and is present in skeletal muscle and the spinal cord. These isoforms may influence microtubule interactions and neurite development; however, little is known about them [151]. Instead of using the 733-amino-acid sequence of big tau, this text refers to tau residues based on the 441-amino-acid sequence of 2N4R tau. The tau protein is encoded by the MAPT gene, located on chromosome 17 at positions q21–22 [152].

Tau function and the risk of developing disease are influenced by several mutations in the coding and noncoding regions of the MAPT gene. More than 60 mutations in the MAPT gene are known to be associated with disease, and many more have been discovered but remain poorly understood [152]. All are thought to enhance function, and no variants that impair function have been identified so far. The majority of tau mutations are linked to FTLD-tau, which presents with symptoms of bvFTD [153]. Mutations in tau’s coding region can affect several properties, including tau’s ability to aggregate, degradation by lysosomal enzymes, interaction with and assembly of microtubules, and susceptibility to phosphorylation through the addition or removal of phosphorylation sites. Some MAPT mutations, such as IVS10+16, exhibit symptoms resembling PSP or CBD, which are usually considered sporadic conditions [154]. In PSP-associated MAPT mutations, exon 10 is more frequently included, increasing the synthesis of the 4-repeat (4R) tau isoform. This results in a 4-repeat tauopathy [155].

Tau SNP analysis has shown that the MAPT gene has two major extended haplotypes, H1 and H2. A 900 kb segment of chromosome 17q21 contains an ancestral inversion that gives rise to these haplotypes. This segment includes at least five other genes in addition to the MAPT gene [156]. In individuals with PSP, CBD, FTD, and Parkinson's disease, the H1 haplotype is far more common than the less frequent H2 haplotype, which may provide protective effects against these disorders [156].

The role of tau haplotypes in Alzheimer's disease (AD) remains unclear. The H1 haplotype appears to increase disease risk only in individuals who do not carry the ApoE4 allele, a common genetic risk factor for AD [157]. Over time, several sub-haplotypes have been identified within H1, prompting investigations into potential associations between disease and other genes in the haplotype [158]. Different expression levels of the LRRC37A gene are associated with a specific H1 sub-haplotype that confers an increased risk for developing Parkinson's disease, as shown in Fig. (**3**). This change may not even be directly related to the MAPT gene [158].

The most compelling evidence supporting a role for tau in brain degeneration comes from the presence of various MAPT mutations. It is important to note that Alzheimer's disease (AD) has never been linked to a tau mutation [159]. Families with dominantly inherited AD have been found to carry several mutations in the presenilin genes (PSEN1 and PSEN2) and in the amyloid precursor protein (APP). The primary reason tau is believed to play a role in AD is that tau accumulation sites detected in imaging and autopsy studies correlate more closely with symptom development than amyloid deposits [160].

Furthermore, a clinical amnestic condition resembling Alzheimer's disease can rarely be induced by two tau mutations, R406W and V337M, which produce 3R:4R tau similar to Alzheimer's disease but without beta-amyloid accumulation [3]. Ablation of the tau gene eliminates Alzheimer's disease symptoms in mouse models of the disease. The optimal timing for intervention in Alzheimer's disease may be the key question, rather than tau's involvement in the condition [161]. Following gene expression and protein synthesis, these proteins can undergo several changes that affect their processing, subcellular localization, and functional activity. This also applies to tau protein [162]. Post-translational modifications (PTMs) such as phosphorylation (tau has more than 60 possible sites), acetylation, glycosylation, O-GlcNAcylation, and cleavage all influence tau function. This has been thoroughly studied in a reference briefly mentioned below [163].

Hyperphosphorylated tau accumulation is the most well-studied type of change, as it is a key marker of pathogenesis in many tau-associated diseases. The amino acids tyrosine, serine, and threonine can all undergo phosphorylation [164]. This process imparts a negative charge to the protein, which can alter its structure and function. Tau's affinity for microtubules is affected by phosphorylation within the microtubule-binding region, and phosphorylation at specific sites may promote tau aggregation [164]. The proline-rich region is a particular area where phosphorylation sites are found in several tau clinical biomarkers; however, the mechanisms driving this phosphorylation are still not well understood [164].

Tau aggregation is inhibited by the addition of a phosphate group at S305; the S305N MAPT mutant lacks this modification. Several innovative medications are being investigated to prevent excessive tau protein phosphorylation. These include GSK3β and Fyn kinase inhibitors, which will be discussed in more detail in a later section [165]. New treatments are also being developed to target acetylation and O-GlcNAcylation. In disease, tau is marked by both an excess of acetyl groups and phosphate groups. Acetylation of tau enhances the aggregation of 3R tau and promotes the formation of tau fibrils [166]. Moreover, acetylation facilitates tau release from the cell, preventing its degradation by chaperone-mediated autophagy [167]. O-GlcNAcylation refers to the attachment of N-acetylglucosamine to serine and threonine residues, which prevents their phosphorylation. O-GlcNAcylation of tau is reduced in brains affected by Alzheimer's disease [168].

The recognition of some post-translational modifications is important even though there are currently no therapeutic strategies targeting them. Glycosylation is the process by which a carbohydrate is attached to a protein [169]. It is the most common post-synthesis modification in proteins. Tau proteins in Alzheimer's disease show abnormal levels of sugar attachment, which may promote phosphorylation. Another notable modification of tau is tau cleavage [170].

Multiple sites of tau cleavage can generate different fragments during disease; both N-terminal and C-terminal fragments are harmful. The protein's C-terminal region contains the domain associated with aggregation and disease. Consequently, fragments truncated at this terminal may have a higher tendency to assemble [171]. In contrast, N-terminal fragments are released into the cerebrospinal fluid, where they exhibit synaptotoxicity. Most biomarker development has focused on their phosphorylation [170]. The observation that different tauopathies can display distinct post-translational modifications (PTMs) is an important finding. This presents both challenges and opportunities for the development of biomarkers and therapies [170]. The identification of specific types of modifications may facilitate the creation of targeted biomarkers or treatments, although the use of some medications for tau-related conditions may be complicated [92].

### 6.1 Tau-based Approach

#### 6.1.1 Reduce Tau Gene Expression (Gene Therapy)

RNA-targeted therapeutics have opened novel options for the treatment of genetic disorders. Reduced tau gene expression may be a promising strategy for treating tauopathies, based on recent advances in antisense oligonucleotides (ASOs) for conditions such as Huntington's disease and spinal muscular atrophy. Because tau serves many functions in the human brain, scientists are cautious about completely eliminating it [172]. However, complete tau ablation does not appear to cause noticeable problems in many mouse studies. This suggests that lowering tau levels could be relatively safe. Research indicates that reducing tau may also provide protection against seizure activity in preclinical models, supporting these findings.

#### 6.1.2 Anti-sense Oligonucleotides (ASOs)

Based on this rationale, Ionis developed BIIB080 (IONIS-MAPT Rx), an intrathecal antisense oligonucleotide that reduces the expression of the entire tau gene. A 50% decrease in tau mRNA levels with BIIB080 reduced tau aggregation in P301S transgenic mice expressing human tau protein. This also improved reproductive activity, reduced neuronal loss, and slowed hippocampal tissue degradation [173, 174]. Positive initial results have led to a Phase 1/2 trial including 46 individuals with mild Alzheimer's disease. These patients will receive BIIB080 treatment for 36 weeks (NCT03186989) [175].

Several studies suggest that 4R-tau may be a harmful form of tau protein, particularly in conditions such as PSP and CBD. Researchers are developing ASOs to target and reduce 4R-tau levels, although this effort remains in preclinical stages [176].

RG6042, a drug developed by Roche and Ionis to reduce mutant huntingtin levels, was evaluated in a clinical trial for Huntington's disease. The results showed a 60% reduction in mutant huntingtin in cerebrospinal fluid [177]. The caudate and other subcortical regions decreased by 20% to 50%, while the cortex decreased by 55% to 85%. The distribution of ASOs in the brain may affect their effectiveness in tauopathies; as observed in PSP, they are more effective in cortical regions than in the caudate, putamen, and thalamic nuclei. RG6042’s Phase 3 trial will evaluate whether reducing mutant huntingtin (mHTT) leads to improved clinical outcomes [177].

#### 6.1.3 Modulate Tau Post-translational Modification (PTM)

Tau can undergo several changes after synthesis, such as the addition of phosphate, acetyl, methyl, and other groups, or by being cleaved into shorter fragments. More than 90 phosphorylation sites are known, along with numerous potential sites for other modifications [178]. With almost limitless variations possible within a single tau molecule, these changes can occur in any of the six tau isoforms present in the brain. Identifying the specific modifications in tau that cause its harmful effects is challenging [119]. Essentially, post-translational modifications (PTMs), particularly phosphorylation, can impair tau’s binding to microtubules, increasing the likelihood of tau misfolding and aggregation [146]. Moreover, studies show that elevated tau phosphorylation is associated with disease progression, and phospho-tau antibodies are available, indicating that tau contributes significantly to disease through phosphorylation [179].

Phosphorylation is neither required nor sufficient for tau-related neuronal damage, according to current data. Scientists suggest that tau hyperphosphorylation is a primary contributor to tau-related problems [180]. Since kinases are commonly targeted in cancer therapy development, they were once a major focus for tauopathy treatments. Drug screening was relatively straightforward, leading to the development and human testing of many kinase inhibitors [180]. In Alzheimer's disease (AD), several studies link protein kinases, such as glycogen synthase kinase 3 beta (GSK-3β), Fyn, and Abl, to harmful tau phosphorylation. However, producing safe and effective kinase inhibitors for long-term tauopathy therapy remains challenging, even if phosphorylation contributes to damage [181].

Although phosphorylation is important, it is unclear which kinase is primarily responsible or whether multiple kinases act together. Post-translational modifications can influence tau activity by preventing O-GlcNAcylation, the addition of N-acetylglucosamine (GlcNAc) moieties to serine/threonine residues *via* O-GlcNAcase (OGA); this modification can reduce kinase-induced hyperphosphorylation [182]. O-GlcNAcylation levels were found to be 50% lower in the brains of Alzheimer's patients compared to healthy individuals, correlating with increased tau hyperphosphorylation. This suggests that inhibiting OGA and enhancing O-GlcNAcylation could represent a potential therapeutic strategy [183].

#### 6.1.4 O-GlcNAcase Inhibitors

Research suggests that focusing on O-GlcNAcylation could reduce tau hyperphosphorylation. Studies with P301L mutant mice showed that the OGA inhibitor Thiamet-G successfully decreased harmful tau aggregation [184]. Subsequently, in collaboration with Merck, Alectos Therapeutics developed the small-molecule OGA inhibitor MK-8719, which demonstrated similar results in transgenic mouse models [184]. The use of a novel radiotracer, [18F]MK-8553, in a Phase 1 study with 16 healthy subjects indicated that MK-8719 was well tolerated and effectively engaged the target. Alectos announced the start of clinical trials for MK-8719 in patients with progressive supranuclear palsy (PSP) in 2016; however, Merck discontinued development, and the trials never commenced [185].

ASN120290 (ASN-561) is another OGA inhibitor developed by Asceneuron. Studies in P301S transgenic mice showed that it reduced phosphorylated tau levels while more than doubling O-GlcNAcylated tau concentrations. Results from a Phase 1 study with 61 healthy participants in 2018 demonstrated that it was well-tolerated and safe [186]. ASN120290 has been designated as an orphan drug. Asceneuron announced in July 2018 that Phase 2 clinical trials for PSP would begin, although these trials have not yet started [187].

#### 6.1.5 Tau Aggregation Inhibitors

In a Phase 2 clinical study, 321 patients with mild to severe Alzheimer's disease were evaluated for methylene blue, which TauRx Therapeutics later renamed Rember. After receiving a moderate dose of 138 mg per day for 24 weeks, cognitive function improved. However, no benefits were observed at the highest dose, and further development of the drug was discontinued [173]. A simpler version of methylene blue, called LMTM (also known as LMT-X or TRx0237), was subsequently developed. A few limited studies have been conducted in healthy participants, and this compound exhibited unique behavior in animal tests [188].

Two other Phase 3 trials, one involving almost 1,700 patients with mild Alzheimer's disease and another involving 220 patients with behavioral variant frontotemporal dementia (the largest trial for this group), showed no benefit from the 200 mg dose of LMTM. The drug demonstrated no improvement over placebo in the primary outcomes [189]. Colorants such as methylene blue and its derivatives can turn urine and feces blue. To maintain blinding, previous studies administered a low amount of the active ingredient (8 mg daily) in placebo groups [189]. The potential efficacy of this low dose, which could have masked earlier findings, prompted the development of a unique active placebo. A Phase 3 trial called LUCIDITY (NCT03446001) is currently evaluating a low dose of LMTM in 450 individuals with early-stage Alzheimer's disease [190].

### 7 ALPHA-SYNUCLEIN: PRECISION MEDICINE STRATEGIES

The synuclein family, which also includes beta and gamma synucleins, contains alpha-synuclein. Alpha-synuclein (AS) is a compact protein of 140 amino acids, primarily found in the central nervous system, with a molecular weight of 14 kDa. It is synthesized by the SNCA gene and carries a strong charge. Predominantly located at presynaptic terminals, alpha-synuclein interacts with synaptic vesicles [191].

In cerebrospinal fluid, AS constitutes about 1% of all proteins. At the N-terminal region of AS, there is a segment that is both hydrophilic and hydrophobic. This region contains eleven structural elements that form seven similar motifs. It can adopt an alpha-helical structure to facilitate lipid binding, which may promote aggregation [192]. The C-terminal region contains the non-amyloid component (NAC), which facilitates calcium binding and modulates protein aggregation. Human alpha-synuclein's NMR structure, as described by Ulmer and colleagues, shows that amino acids 3–37 and 45–97 form curved alpha-helical regions [193]. These regions are arranged in an antiparallel fashion and are connected by an extended segment. Amino acids 98–140 constitute a highly flexible region. The structured configuration of the helix connector enhances lipid surface compatibility, allowing it to act as a bridge between larger synaptic vesicles and a continuous helical model proposed previously [194]. Although its physiological role is not fully understood, AS is associated with disorders such as multiple system atrophy, dementia with Lewy bodies, and Parkinson's disease [195].

These dimers may aggregate in different ways due to their similar structures. The homozygous SNCA p.A53V mutation causes a distinct phenotype characterized by progressive Parkinsonism and cognitive decline, similar to that observed with other SNCA missense mutations, according to a mutation-frequency analysis in Japanese patients [196]. AS interacts more readily with synaptic vesicles attached to the plasma membrane due to its disrupted helix structure, which consists of two antiparallel membrane-bound helices connected by a non-helical linker [197].

Tyrosine at position 39 (Y39) in AS has been shown in laboratory studies to undergo phosphorylation, causing the protein to detach from the vesicular membrane. This occurs because, in a degraded state, helix-2’s attachment is reduced. This effect is comparable to that of the G51D mutant [198]. The beta-hairpin structure of peptide 1a, which contains residues 36–55 of AS, subsequently aggregated into a triangular trimer. Molecular modeling results suggest that a full assembly of AS might be constructed [198]. Additionally, this 1a peptide demonstrated the ability to bind anionic lipid bilayer membranes and promote AS oligomerization [199]. A study of 426 Parkinson’s disease patients in Italy found that individuals with a specific genetic variant, the 263 bp allelic variant of Rep-1, were more likely to experience dementia and hallucinations [199]. This variant is located near the start of the SNCA gene. Compared to individuals without it, carriers of this variant are more likely to exhibit these symptoms [196].

Research has shown that Chinese individuals with Parkinson's disease (PD) exhibit reduced resting brain activity in certain regions, particularly the left caudate and lingual gyrus. This was determined by comparing their brain function to that of healthy individuals [200]. ALFF scores in the right fusiform region were lower in those carrying the G allele of the rs894278 variant within the SNCA gene than in non-carriers. This study suggests that Parkinson's disease may alter the functioning of neuronal connections in the brain [200]. The etiology of sporadic Parkinson's disease may involve specific genetic variants in SNCA in addition to known mutations. Evidence links SNPs rs7684318, rs894278, and rs2572324 to an increased risk of developing Parkinson's disease independently [201].

Some genes implicated in Parkinson's disease may also contribute to vitiligo. Neuroinflammation plays a role in the loss of dopaminergic neurons. It involves the immune system attacking melanocytes, potentially contributing to their destruction and leading to loss of pigmentation. Intracellular signaling pathways, including oxidative stress, the JAK/STAT pathway, and the Type I IFN pathway, show potential overlap with Parkinson's disease [202].

Additionally, individuals with sporadic Parkinson's disease have been found to carry missense mutations A29S and A18T. SNPs rs2736990 and rs356220 were linked to an increased risk of sporadic Parkinson's disease in a Japanese population study. Several genetic alterations, as well as other unknown biological factors, induce alpha-synuclein (AS) to misfold into beta-sheet-rich amyloid fibrils [203]. Tuttle and colleagues determined the solid-state NMR structure of these destructive amyloid fibrils. More than 200 distinct long-range distance constraints are present in AS fibrils, which help define a common structural arrangement [204]. This structure is composed of various elements, including a glutamine ladder, intermolecular salt bridges, hydrophobic core regions, aligned beta sheets, small residues that allow tight backbone interactions, and other features that maintain overall stability [204]. Since amyloid fibrils can exist in multiple viable forms, they are typically polymorphic. Guerrero-Ferreira and colleagues identified two new AS fibril types with distinct morphologies [205].

The variability in drug response is related to its pharmacokinetic and pharmacodynamic properties. In neurological diseases, the blood-brain barrier (BBB) is also a crucial factor regulating the intra-cerebral concentration of the drug [206]. Moreover, genetic variations at the DNA or RNA level can directly or indirectly alter the expression or activity of proteins involved in the drug’s mechanism of action or metabolism. These parameters may be influenced by multiple factors, including environmental factors, drug interactions, and pathological or physiological conditions, as well as genetic variability [206].

An overabundance of alpha-synuclein (AS) may disrupt the body’s calcium balance, which AS helps regulate, increasing the risk of dopaminergic neuron damage. Some studies suggest that AS prefers curved membranes and specific membrane regions, interacting effectively with phospholipid membranes, particularly those of synaptic vesicles [207]. AS regulates postsynaptic terminal size and synaptic vesicle positioning. The alpha helix at the N-terminus is essential for lipid binding [207]. AS also affects the SNARE protein complex, potentially lowering dopamine and glutamate levels. Excess AS can delay vesicle maturation, weaken synaptic connections, and hinder vesicular transport *via* the endoplasmic reticulum and Golgi apparatus. The normal form of AS can interfere with vesicle endocytosis and disrupt neuronal transmission when present in excess [208].

Alpha-synuclein (AS) may contribute to Parkinson’s disease by altering dopamine synthesis, storage, reuptake, reutilization, and excretion. Elevated AS levels have been shown to reduce the amount of active tyrosine hydroxylase (TH), which is essential for dopamine synthesis. Overexpression of AS has also been observed to impair vesicular monoamine transporter 2 (VMAT2) function [209]. VMAT2 normally protects against oxidative damage by sequestering dopamine in synaptic vesicles after its synthesis. When AS reduces VMAT2 activity, increased cytoplasmic dopamine levels may become toxic and contribute to Parkinson’s disease pathology [210]. Dopamine transport within the brain is mediated by the dopamine transporter (DAT); however, whether AS increases or decreases DAT levels remains controversial, with evidence supporting both possibilities [211].

Although there are currently no proven cures for Parkinson’s disease, medications and surgical interventions can improve motor function and alleviate symptoms. Targeting alpha-synuclein (AS), a key contributor to PD, represents an effective strategy to mitigate its pathological effects [212]. Several therapeutic approaches have recently been developed to reduce AS-associated toxicity. Propagation can be controlled by blocking AS receptors; for example, antibodies targeting LAG3 have been shown to lessen the detrimental effects caused by abnormal AS [212]. Additionally, shRNA and siRNA have been employed to suppress AS expression in rat and mouse brain models [213]. Small molecules such as Anle138b can inhibit AS oligomer formation and accumulation, while other chemical inhibitors, including methylthioninium, have effectively controlled AS fibrillar inclusions in both *in vitro* and *in vivo* models [213]. Moreover, various plant extracts and phytochemicals have demonstrated potential in modulating AS aggregation in PD models, reflecting growing interest in botanical strategies to target amyloid-related pathologies.

## 8 INTERPLAY OF PROTEINS AND GENES IN PD PATHOGENESIS

Parkinson’s disease arises from defects in three critical cellular mechanisms: synaptic vesicle endocytosis, mitochondrial function, and lysosomal and proteasomal activity [214-221]. Neuronal damage from oxidative stress and the accumulation of damaged or misfolded proteins can impair these pathways. The disease manifests when an excess of mutant proteins overwhelms the protective mechanisms of dopaminergic neurons [222]. Mitophagy, the process responsible for removing dysfunctional mitochondria, is facilitated by PINK1. This protein either acts independently or recruits Parkin to repair damaged or malfunctioning regions of the mitochondrial membrane. The PINK1/Parkin pathway is also essential for proper mitochondrial fusion and fission events [223].

Damaged mitochondria may accumulate within cells due to mutations or deficiencies in key proteins, leading to oxidative stress. Mitochondrial-derived vesicles (MDVs) are specialized structures that transport oxidized proteins from mitochondria to lysosomes during oxidative stress caused by reactive oxygen species [224]. The PINK1/Parkin pathway may also influence this process. In neurons, DJ-1 detects oxidative stress and can enter damaged mitochondria, providing additional protection against stress-induced apoptosis [224]. Mutations in DJ-1 are thought to reduce autophagy and lysosomal activity. Mutations, gene duplication, or triplication that increase alpha-synuclein (AS) levels can further induce oxidative stress and mitochondrial dysfunction [225].

Glucocerebrosidase, encoded by the GBA gene, has been proposed to degrade AS in lysosomes and reduce its toxicity, while also converting glucosylceramide into membrane constituents. Mutations in GBA have been shown to cause lysosomal-autophagy dysfunction, oxidative stress, mitochondrial fragmentation, impaired ubiquitin-proteasome activity, and altered lipid metabolism [226]. Similarly, mutant LRRK2 has been linked to oxidative stress-induced apoptosis, disrupted vesicular trafficking, and accumulation of misfolded proteins [227]. Recent studies indicate that neurons derived from patients with mutant PINK1 expression exhibit elevated LRRK2 mRNA and protein levels, suggesting that PINK1 regulates LRRK2 levels in neurons. DJ-1 is also thought to modulate PINK1 and AS levels in neuronal cells [228].

## 9 GENOMIC APPROACH USING CRISPR-CAS9

### 9.1 SNCA — Synuclein Alpha (SNCA) Gene

Transplanted human fetal brain cells have been observed to develop Lewy bodies in clinical trials involving patients with Parkinson's disease. To address this potential limitation, CRISPR-mediated deletion of the SNCA gene in human embryonic stem cells has been employed to generate engineered dopaminergic neurons resistant to synucleinopathy [229]. In this study, SNCA^+/− and SNCA^−/− neurons exposed to pre-formed α-synuclein fibrils showed no evidence of protein aggregation. In a separate study, SNCA expression was successfully reduced using a specialized lentiviral vector designed to selectively modify DNA methylation [230].

The use of iPS cells in Parkinson's disease models has shown considerable promise. To enhance this approach, a successful method for generating biallelic genome-edited populations using CRISPR-Cas9 has been developed [231]. This technique, called FACE, combines CRISPR-Cas9 editing with FACS and employs a fluorescent marker to facilitate the generation of precisely modified cells while eliminating unwanted or incorrectly edited cells [231]. To model Parkinson's disease *in vitro*, a panel of human SNCA mutations was introduced. These isogenic lines carry p.A30P or p.A53T mutations in the SNCA gene, which are associated with the disease [232]. Fig. (**4**) illustrates CRISPR-Cas9 targeting of the SNCA gene to reduce Lewy body formation and mitigate neurodegenerative pathology.

### 9.2 GBA — Glucosylceramidase Beta (GBA) Gene

Hereditary and sporadic Parkinson's disease, as well as other α-synuclein-related disorders, are associated with the aggregation and misfolding of α-synuclein. The precise mechanism by which α-synuclein tetramers are formed remains unclear [233]. Systematic studies using CRISPR-mediated GBA1 knockout have shown that mutations in glucosylceramidase beta 1 (GBA1) and the resulting deficiency in glucocerebrosidase destabilize α-synuclein tetramers. This increases the vulnerability of human dopaminergic neurons to cytotoxicity induced by pathological α-synuclein fibrils [234]. Their findings suggest that correcting cholesterol imbalances could provide new therapeutic opportunities for Parkinson's disease (PD).

### 9.3 LRRK2

Induced pluripotent stem (iPS) cells derived from individuals with Parkinson's disease (PD) were subjected to CRISPR/Cas9 genome editing to generate isogenic PD astrocytes and ventral midbrain dopaminergic neurons lacking the LRRK2 G2019S mutation [235]. Experiments with PD astrocytes and dopaminergic neurons revealed atypical increases in α-synuclein protein and signs of cellular damage, resulting from impaired lysosomal degradation of α-synuclein [236]. Conversely, co-culturing PD ventral midbrain dopaminergic neurons with healthy astrocytes prevented the emergence of disease-related phenotypes [236]. In these cells, macroautophagy was shown to be significantly affected. These findings indicate that dysfunctional astrocytes play a critical role in the pathophysiology of Parkinson's disease [237]. Additionally, dCas9, dCas9-KRAB, and dCas9-VPR can be used to target the transcriptional initiation sites of SNCA, MAPT, HTT, and APP to block their expression, providing a means to determine the timing and cause of Parkinson’s disease and other neurological disorders [238]. Specific CRISPR/Cas9 strategies also enable precise SNCA modifications, facilitating detailed investigations into disease mechanisms.

## 10 CHALLENGES IN TRANSLATING GENOMIC APPROACHES INTO CLINICAL PRACTICE

Precision medicine offers a novel approach to treating neurological disorders such as Parkinson's and Alzheimer's disease by targeting key molecules, including alpha-synuclein, tau, and amyloid-β (Aβ) [239]. Advances in whole-genome sequencing, transcriptome analysis, and other genetic tools have identified specific alterations in genes such as APP, PSEN1, MAPT, and SNCA that directly influence disease mechanisms [239]. For example, mutations in APP and PSEN1 can lead to excessive production of toxic Aβ protein, causing familial Alzheimer's disease, whereas changes in the MAPT gene can increase susceptibility to tauopathies by affecting tau protein stability and expression [239]. Similarly, alterations in the SNCA gene promote alpha-synuclein aggregation, contributing to Parkinson's disease [240].

Building on these findings, researchers are developing novel genetic strategies, including CRISPR-based gene editing, to treat hereditary disorders, reduce toxic protein levels, or modulate protein aggregation. The use of polygenic risk assessment and single-cell RNA sequencing allows for patient stratification based on individual genetic and molecular profiles, facilitating the development of highly personalized therapeutics [241].

Despite these promising advances, significant challenges remain in implementing genetics-based precision medicine. Chief among these is the complex interplay between genes and environmental factors in neuronal degeneration. While gene mutations, such as those in APOE or LRRK2, represent important risk factors, their effects vary considerably across individuals and populations [242].

This complexity makes it more difficult to predict the onset or progression of neurodegenerative diseases. Given that these disorders involve multiple pathological features, targeting a single molecule may be insufficient [243]. Patients often exhibit overlapping abnormalities in alpha-synuclein, tau, and amyloid-β. Access to advanced genomic testing and therapies is further limited in low-resource settings due to high costs and the specialized expertise required [243]. Concerns regarding genetic modifications, long-term safety, and equitable access also hinder the widespread adoption of these novel treatments. Integrating genetic research with robust clinical trials, empirical evidence, and supportive policy frameworks is essential to ensure that new therapies are both effective and accessible to a broad population [244].

## 11 KEY RISKS AND LIMITATIONS OF GENOMIC THERAPY

### 11.1 Off-target Effects

The very high frequency of off-target effects (OTEs), reported at≥50%, is a significant issue when using CRISPR/Cas9 for gene therapy [245]. Optimizing guide designs and creating Cas9 variations with lower OTE are two current efforts to allay this worry. Cas9 nickase (Cas9n), a variation that causes single-stranded breaks (SSBs), is combined with an sgRNA pair that targets both strands of the DNA at the desired location to create the DSB, which is one method that reduces OTEs [246]. Additionally, scientists have created Cas9 variations that are specially designed to lower OTEs without sacrificing editing effectiveness. One of these high-fidelity variations, SpCas9-HF1, utilizes the “excess-energy” theory, which postulates that Cas9 and target DNA have an excess affinity that might facilitate OTEs [246]. Many of these techniques are based on computational methods with variable parameters or rely on phenotypic screens that could be unique to distinct cell types and genomes. As a result, they produce a significant amount of noise and are not generally applicable to other experimental settings [247].

### 11.2 DNA-Damage Toxicity

Instead of causing the desired gene change, CRISPR-induced DSBs frequently cause apoptosis. Using this method in human pluripotent stem cells (hPSCs) raised further safety concerns since it showed that p53 activation in response to the harmful DSBs created by CRISPR frequently results in eventual apoptosis [248]. Therefore, p53-suppressed cells are more likely to undergo effective CRISPR edits, which leads to a bias favoring the survival of oncogenic cells. A significant safety concern for clinical uses of DSB-inducing CRISPR treatment is highlighted by the numerous reports of massive deletions spanning kilobases and complex rearrangements as unintended outcomes of on-target activity [249].

### 11.3 Delivery of CRISPR Gene Therapy

The safety and therapeutic efficacy of CRISPR tools are strongly influenced by their delivery method. Adeno-associated virus (AAV) vectors remain a key system for CRISPR gene therapy due to their high delivery efficiency, even though conventional viral gene therapy has raised concerns about immunotoxicity and insertional oncogenesis [250]. Components of the CRISPR toolkit, such as Cas9 and gRNA, can be delivered as mRNA or packaged as plasmid DNA [250]. CRISPR nucleic acids can be introduced *via* AAV vectors or delivered directly to target cells through microinjection or electroporation/nucleofection, which bypass the risks associated with viral delivery [250]. Microinjection, however, is limited to *ex vivo* applications and can be technically challenging. Electroporation is commonly used *ex vivo*, though it can also be applied *in vivo* for certain tissues; the high-voltage shock required can damage cells and render membranes permanently permeable [250]. Despite these limitations, current delivery systems have enabled clinical applications of CRISPR gene therapy, with ongoing improvements continuing to enhance their safety and effectiveness [250].

## 12 ETHICAL IMPLICATIONS OF USING GENE EDITING

When a person is thinking about getting genetic testing that will reveal genetic information about them and their family members, ethical considerations are crucial. When it comes to the ethical ramifications of genetic testing for neurodegenerative illnesses, medical practitioners play a crucial role. However, a clinician's judgment frequently determines the usage and ethical consideration of genetic testing in treatment. The choice was made without the assistance of thorough study evidence. Finding appropriate volunteers with certain neurological diseases can be challenging, making patient recruitment a major study hurdle. Furthermore, the development of neurological illnesses varies greatly from person to person, making it difficult to define consistent trial endpoints. Given the fragility of many patients and the complexity of obtaining informed consent due to cognitive impairments, ethical issues are crucial. Regulatory obstacles increase complexity, which must be overcome to guarantee the safety of experimental medicines while accelerating approvals for life-threatening illnesses. Lastly, because many neurological illnesses are chronic, they require extensive follow-up periods, which can be costly and logistically challenging. Resolving these issues is essential to developing successful therapies for people with neurological conditions.

## 13 FUTURE DIRECTIONS IN PRECISION MEDICINE FOR NEURODEGENERATIVE DISEASES

In precision medicine for neurodegenerative diseases, personalized treatments are developed by integrating genetic and biochemical data to target key disease pathways, including tau, alpha-synuclein, and amyloid-β. Recent genome sequencing has identified numerous genetic variants associated with specific diseases. Amyloid-β accumulation in Alzheimer's disease is linked to mutations in the APP, PSEN1, and PSEN2 genes [251]. Misfolding of alpha-synuclein in Parkinson's disease is caused by mutations in the SNCA gene, whereas tau abnormalities in frontotemporal dementia result from MAPT mutations. These discoveries have enabled the development of targeted therapies, such as antibodies against amyloid-β (aducanumab and lecanemab) and tau aggregation inhibitors (tilavonemab), as well as small molecules targeting alpha-synuclein, such as anle138b. Genomic approaches also facilitate the identification of biomarkers for predicting treatment response, monitoring disease progression, and accelerating diagnosis [252].

Tools like transcriptomics and polygenic risk scores allow for the identification of discrete patient subgroups. Therapeutics such as tofersen for SOD1-linked ALS and CRISPR/Cas9 gene editing show promise in altering disease trajectories through precise genetic interventions. Advancing precision medicine for neurodegenerative diseases will require multi-omics strategies, including genomics, epigenomics, proteomics, and metabolomics, to construct complex disease networks and uncover novel therapeutic targets. Machine learning and artificial intelligence will play a critical role in analyzing large datasets, enhancing drug discovery, and optimizing personalized treatment strategies [253].

Other emerging technologies include induced pluripotent stem cells (iPSCs) derived from the patient's cells, which enable the *in vitro* simulation of neurodegenerative diseases to accelerate the development of tailored treatments. By enhancing precision treatment for often marginalized groups, research into the genetic makeup of certain communities will help to reduce healthcare inequities. Neurodegenerative diseases can be remotely monitored in real-time with new wearable and digital health gadgets. This enables timely adjustments to treatment plans when needed. Preventive precision medicine, which involves identifying at-risk individuals through genetic testing before any symptoms appear, is the goal. Our approach to neurodegenerative diseases will change as a result [6].

## CONCLUSION

This review highlights promising advances in precision medicine for neurodegenerative diseases, emphasizing the critical role of key proteins in conditions such as Alzheimer's and Parkinson's through genetic and genomic approaches. Significant progress in understanding the genetic and molecular mechanisms underlying these diseases has enabled the development of targeted therapies, including antibodies and small molecules directed against amyloid-β, tau, and alpha-synuclein. The review also addresses ongoing challenges in translating these findings into clinical practice, including the complexities of gene–environment interactions, the need for multifactorial treatment strategies, and barriers related to access and cost. The authors advocate for continued interdisciplinary research and collaboration to optimize therapeutic interventions. They suggest that integrating genetic insights with advanced technologies and clinical applications could pave the way for more effective, personalized treatments for neurodegenerative disorders. Ultimately, the aim is to improve patient outcomes and reduce the growing burden of these diseases.

## Figures and Tables

**Fig. (1):**
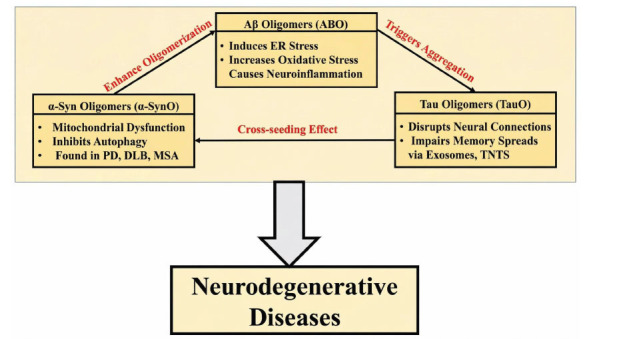
Aβ, Tau, and α-Syn interactions in neurodegenerative diseases.

**Fig. (2):**
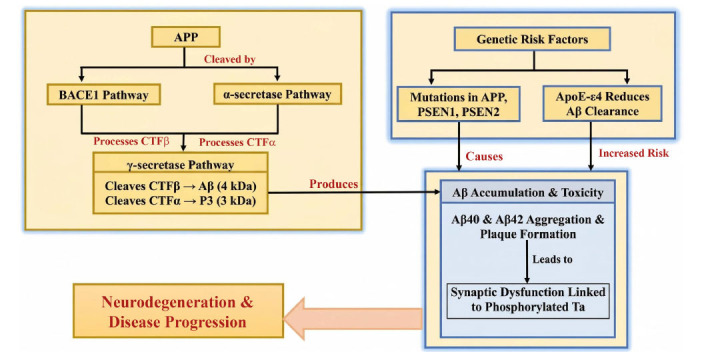
APP processing pathways and genetic risk factors contributing to Aβ accumulation and neurodegeneration.

**Fig. (3):**
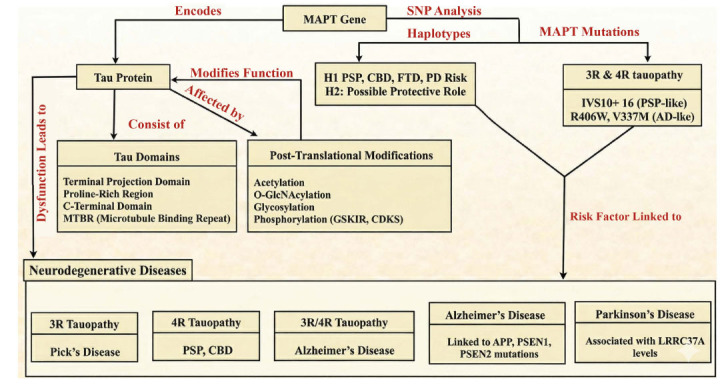
Role of MAPT gene mutations and tau protein dysfunction in neurodegenerative disease.

**Fig. (4):**
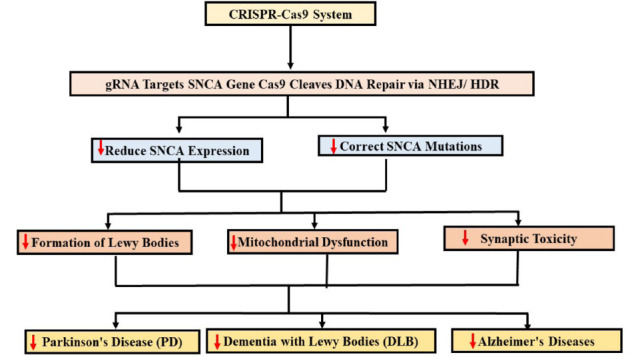
CRISPR-Cas9 targeting of SNCA gene to reduce Lewy body formation and mitigate neurodegenerative diseases.

**Table 1:** Aptamer treatments targeting neurodegenerative disorders for precision medicine. Roles and therapeutic benefits.

**Neurodegenerative Disorder**	**Aptamer** **Target**	**Mechanism of Action**	**Precision Medicine Approach**	**Current Research/Trials**	**Potential Benefits**	**References**
**Alzheimer’s Disease (AD)**	Beta-amyloid (Aβ)	Inhibits Aβ aggregation	Patient-specific biomarker targeting	Preclinical studies on Aβ-targeting aptamers	Reduced plaque formation, early diagnosis	[79, 80]
**Parkinson’s Disease (PD)**	Alpha-synuclein	Blocks toxic fibril formation	Targeted therapy based on genetic risk factors	Clinical trials for synuclein-targeting aptamers	Slows neurodegeneration, personalized therapy	[81-83]
**Huntington’s Disease (HD)**	Mutant huntingtin (mHTT)	Selective binding and degradation	RNA-based aptamers for allele-specific therapy	Early-stage research on RNA aptamers	Prevents mutant protein accumulation	[84, 85]
**Amyotrophic Lateral Sclerosis (ALS)**	SOD1, TDP-43	Reduces misfolded protein toxicity	Precision-targeted molecular therapy	*In vitro* studies using aptamers against TDP-43	Delays disease progression	[86-89]
**Multiple Sclerosis (MS)**	Myelin oligodendrocyte glycoprotein (MOG)	Blocks autoantibody binding	Personalized treatment based on immune profiling	Aptamer-based immunomodulation under study	Reduces inflammation and demyelination	[90]
**Frontotemporal Dementia (FTD)**	Tau protein	Prevents tau aggregation	Targeted therapy for genetic subtypes	Preclinical studies on tau-targeting aptamers	Neuroprotection and cognitive improvement	[91, 92]
**Lewy Body Dementia (LBD)**	Alpha-synuclein	Inhibits oligomer formation	Personalized medicine based on Lewy body pathology	Research on aptamer specificity	Improved cognitive and motor symptoms	[93-95]
**Spinal Muscular Atrophy (SMA)**	SMN protein	Enhances SMN protein levels	Gene-specific aptamer therapeutics	Preclinical studies on SMN-targeting aptamers	Improved motor function and lifespan	[96, 97]
**Stroke-induced Neurodegeneration**	Thrombin	Anticoagulation and neuroprotection	Individualized therapy for stroke patients	Aptamer-based thrombin inhibitors in trials	Reduced secondary brain injury	[98-100]

## LIST OF ABBREVIATIONS

ALS: Amyotrophic Lateral Sclerosis
AMD: Age-Related Macular Degeneration
AMPK: AMP-Activated Protein Kinase
APP: Amyloid Precursor Protein
Asos: Antisense Oligonucleotides
Aβ: Amyloid-Beta
Aβo: Amyloid Β Oligomers
Cdk5: Cyclin-Dependent Kinase 5
CRISPR: Clustered Regularly Interspaced Short Palindromic Repeats
DSB: Double-Strand Break
ER: Endoplasmic Reticulum
GWAS: Genome-Wide Association Studies
HDR: Homology-Directed Repair
Ipscs: Induced Pluripotent Stem Cells
MAPT: Microtubule-Associated Protein Tau
NGS: Next-Generation Sequencing
NHEJ: Non-Homologous End Joining
POLR: Polymerase
PSEN1: Presenilin 1
RNA: Ribonucleic Acid
SNCA: Synuclein Alpha
TDP-43: TAR DNA-Binding Protein 43
WES: Whole Exome Sequencing
Α-Syn: Α-Synuclein
